# High Molecular Weight Glucan of the Culinary Medicinal Mushroom *Agaricus bisporus* is an α-Glucan that Forms Complexes with Low Molecular Weight Galactan

**DOI:** 10.3390/molecules15085818

**Published:** 2010-08-25

**Authors:** Fhernanda R. Smiderle, Guilherme L. Sassaki, Jeroen van Arkel, Marcello Iacomini, Harry J. Wichers, Leo J.L.D. Van Griensven

**Affiliations:** 1 Plant Research International, Wageningen University and Research Centre, Droevendaalsesteeg 1, 6708 PB Wageningen, Netherlands; 2 Departamento de Bioquimica e Biologia Molecular, Universidade Federal do Paraná, Centro Politécnico, CP 19046, Curitiba-PR, Brazil; 3 Wageningen University and Research Centre, Bornse Weilanden 9, 6708 WG Wageningen, Netherlands

**Keywords:** *Agaricus bisporus*, basidiomycetes, α-glucan, medicinal mushrooms, polysaccharides, NMR

## Abstract

An α-glucan was isolated from the culinary medicinal mushroom *A. bisporus* by hot water extraction, ethanol precipitation and DEAE-cellulose chromatography. The resulting material showed a single HMW peak excluded from a Sephadex G50 column that could completely be degraded by α-amylase treatment. After heating in 1% SDS a small additional peak of low MW eluted from the G50 column. The monosaccharide composition of the main peak was evaluated by HPLC, and was found to consist of a majority of glucose (97.6%), and a minor proportion of galactose (2.4%). Methylation analysis and degradation by α-amylase indicated the presence of an α-glucan with a main chain consisting of (1→4)-linked units, substituted at O-6 by α-D-glucopyranose single-units in the relation 1:8. Mono- (^13^C-, ^1^H-NMR) and bidimensional [1H (obs.),13C-HSQC] spectroscopy analysis confirmed the α-configuration of the Glc*p* residues by low frequency resonances of C-1 at δ 100.6, 100.2, and 98.8 ppm and H-1 high field ones at δ 5.06, 5.11, and 4.74 ppm. The DEPT-^13^C-NMR allowed assigning the non-substituted and *O*-substituted –CH_2_ signals at δ 60.3/60.8 and 66.2 ppm, respectively. Other assignments were attributed to C-2, C-3, C-4, C-5 and C-6 of the non-reducing ends at δ 71.8; 72.8; 70.0; 71.3 and 60.3/60.8 ppm, respectively. The minor proportion of galactose that was demonstrated was probably derived from a complex between the α-glucan and a low molecular weight galactan.

## 1. Introduction

Medicinal mushrooms such as *Agaricus brasiliensis* S. Wasser *et al.* (*= A. blazei* Murrill s. Heinem.), *Coprinus comatus* (O. F. Müll) Pers., *Trametes* (= *Coriolus versicolor* (L.: Fr.) Lloyd, *Ganoderma lucidum* (W. Curt.: Fr.) P. Karst., *Lentinula edodes* (Berk.) Pegler, *Phellinus linteus* (Berk.: Curt.) Teng, and many others have traditionally been used as a health food or supplement for the prevention and cure of a range of diseases, including atherosclerosis, cancer, chronic hepatitis, and diabetes. The preventive and therapeutic effects of these mushrooms and their components have been well documented in mouse and rat model systems and in cancer cell lines [1,2,3,4]. This has led to a considerable amount of knowledge of the effects of mushroom extracts and of their modes of action.

It is generally accepted that mushroom extracts contain a variety of components, such as polysaccharides (*i.e*. glucans), small proteins, lectins and polyphenols, each of which may have its own biological or medicinal effects. The most common immunomodulatory effects of mushroom are attributed to β-(1→3)-(1→6)-glucans, which have been studied in quite some detail. A possible mechanism for their action is via binding to β-glucan-specific receptors on innate immune cells, such as monocytes or macrophages, which may lead to subsequent activation of adaptive immunity, particularly of a Th1-like response (for recent reviews, see e.g., [5,6]). Their potential role to mitigate allergic disease has been reviewed recently [7]. These kind of polysaccharides exist as triple helices in aqueous solution, and several studies suggest that these higher ordered structures are responsible for the mentioned activities [8]. The triple helix conformation also plays an important role in enhancing the antitumor effects of lentinan [9]. When schizophyllan was tested, however, the helical arrangement was not decisive and other factors were found more important. The same molecules were able to stimulate TNF-α activity in U937 cells in a single helical conformation [8,9]. Recently a glycogen-like polysaccharide that potently activated macrophages, stimulating the TNF-α production and phagocytosis in RAW264.7 cells, was isolated [10]. The biological activity of the α-glucans seems to be related to their molecular weight. Glycogens of 14,000-24,000K were not able to promote the release of NO by RAW264.7 cells into the culture fluid, while glycogens of 6,500K strongly stimulated the NO production [11].

The cultivated white button mushroom *Agaricus bisporus* (J. Lange) Imbach was shown to enhance Natural Killer Cell activity in mice, suggestive for potential for promoting innate immunity against tumors and viruses [12]. When tested in hypercholesterolemic rats, the *A. bisporus* extracts decrease the hepatic cholesterol and triglyceride concentrations (36.2% and 20.8%) [13]. Independently we demonstrated that mouse bone marrow derived macrophages could be activated by *A. bisporus* hot water extract, as shown by an increase in nitric oxide production [14]. However, the same extract controversially lowered nuclear factor-kB transactivation in human intestinal CaCo-2 NF-κB reporter cells [15]. Further, *A. bisporus* extracts were shown to contain prooxidative phenolic compounds [16,17] and could inhibit proliferation of HL-60 respectively of K562 leukemia cells by induction of apoptosis [17,18]. Finally, it is reported that orally administered hot water extracts of *A. bisporus* could partially cure streptozotocin-induced diabetes in rat [19].

Since it appears unlikely that all these effects are caused by a single compound, and as some parameters like molecular weight, branching degree, helical conformation and purity can influence their activity, it was deemed imperative to further characterize the contents of *A. bisporus* derived hot water extracts.

In an earlier study [20] we reported the presence of two different classes of polysaccharides in the hot water extract of fruiting bodies of this mushroom. Approximately 70% consisted of a colorless high MW glucan (MW > 200 kDa) that did not bind to DEAE cellulose and showed no pro- or anti-oxidative effects. Acid hydrolysis followed by HPLC analysis showed it to consist of 96.3% glucose residues, with some minor proportions of galactose (2.9%) and xylose (0.8%). The other 30% of the extracted polysaccharides was a hazel colored polysaccharide-polyphenol complex of MW 75-200 kDa that showed strong pro-and anti-oxidative effects. In another study [15] the high MW glucan was subjected to linkage analyses via GC-MS of its partially methylated alditol acetates. This showed that it consisted of a linear glucan with 85.5% 1→4 linkages and only 3.5% 1→3 linkages. Its α-amylase sensitivity suggested an α-configuration of the glucan. In the present study we further characterized the *A. bisporus* high MW glucan and established its definite structure by NMR spectroscopy.

## 2. Results and Discussion

**Figure 1:**
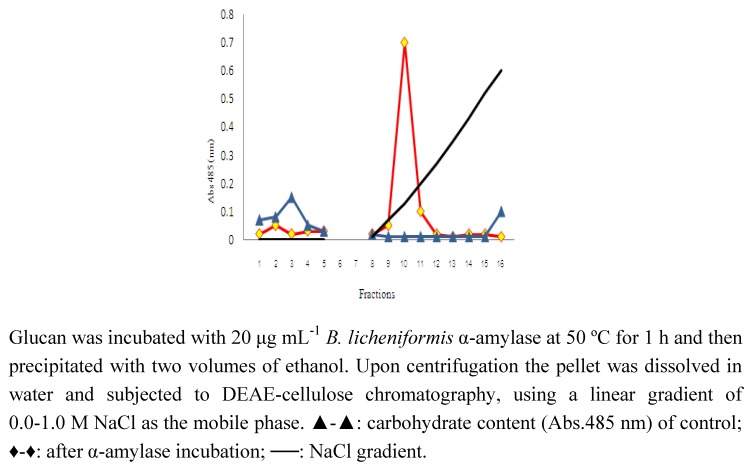
DEAE-cellulose chromatography of high MW glucan of *A. bisporus* before and after incubation with α-amylase.

**Figure 2:**
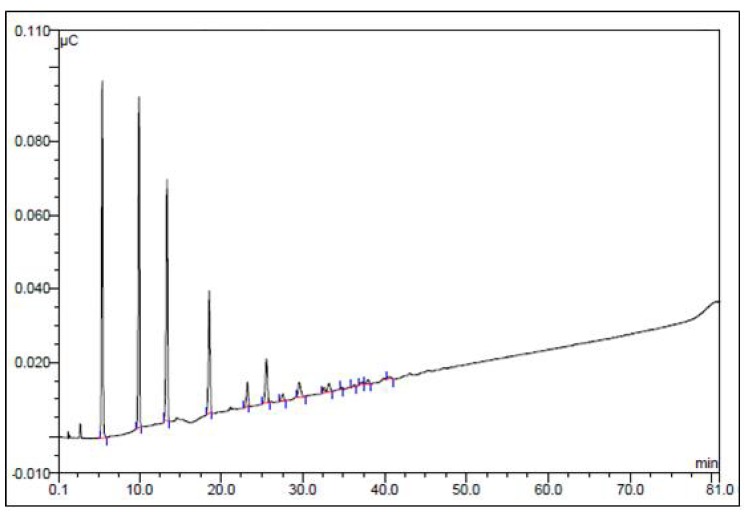
Dionex anion exchange HPLC profile of high MW glucan of *A. bisporus* after incubation with 300 U mL^-1^ α-amylase for 1 h at 50 °C. The glucan was then loaded onto the column and eluted as described in the Experimental.

When the purified high MW polysaccharide from *A. bisporus* was incubated with α-amylase and then reprecipitated with ethanol and subjected to DEAE cellulose column chromatography it was found that none of the material was retained on the column. This indicated that the treated polysaccharide was either degraded into fragments too small to be precipitated by two volumes of ethanol or had not been able to bind to the DEAE cellulose. Figure 1 shows the elution profile of α-amylase-treated polysaccharide and the non-treated control that had been given the same treatment without α-amylase added. Figure 2 shows that approximately 17% of the degradation products consist of monomeric glucose. The majority of the reaction products consist of di-, tri-, tetra-, penta- and larger oligosaccharides in serially decreasing amounts. This is in agreement with the degradation profile of α-amylase-degraded native Oyster glycogen [21]. The sensitivity of this polysaccharide to α-amylase is indicative of the presence of (1→4)-linked α-glucose.

Our earlier observation [20] that this same high MW material had not bound to DEAE-cellulose during its extraction procedure is most likely due to the presence of residual salt in the crude hot water extract of fruiting bodies. Acid hydrolysis followed by GC-MS analysis indicated that this sample consisted of glucose as the major component (90.8%) and galactose in a minor proportion (9.1%) (Table 1).

**Table 1:** Composition of the acid (TFA) hydrolysate of high MW glucan of *A. bisporus*.

Monosaccharides (%)					
**Glucose**	**Mannose**	**Galactose**	**Arabinose**	**Ribose**	**Rhamnose**
90.1	-	9.8	Tr.	Tr.	Tr.

Methylation analyses were carried out and the partially *O*-methylated alditol acetates, analyzed by GC-MS, are shown in Table 2. The main chain of this polysaccharide is composed of α-(1→4)-Glc*p* residues as indicated by the majority of 2,3,6-Me_3_-Glc*p* (72.5%) derivatives, and the presence of 2,3-Me_2_-Glc*p* (8.3%) residue indicates that this molecule is branched at O-6 position. Also 2,3,4-Me_3_-Gal*p* (9.3%) and the non-reducing ends 2,3,4,6-Me_4_-Glc*p* (9.9%) can be seen. The presence of both these derivatives, in this proportion, suggests that the branches are composed of Glc*p* and Gal*p* residues, indicative of the presence of a galactoglucan instead of a glucan.

**Table 2:** Partially *O*-methylalditol acetates formed on methylation analysis of glucan isolated from *A. bisporus*.

Partially *O*-methylated alditol acetates ^(a)^	Mol %	Linkage Type ^(c)^
	Sample ^(b)^	
2,3,4,6-Me_4_-Glc*p*	9.9	Glc*p-*(1→
2,3,6-Me_3_-Glc*p*	72.5	4→)-Glc*p-*(1→
2,3,4-Me_3_-Gal*p*	9.3	6→)-Gal*p-*(1→
2,3-Me_2_-Glc*p*	8.3	4,6→)-Glc*p-*(1→

^a^ Analyzed by GC-MS, after methylation, total acid hydrolysis, reduction with Na_2_B^2^H_4_ and acetylation; ^b^ % of peak area relative to total peak area; ^c^ Based on derived *O*-methylalditol acetates.

To identify whether this fraction was a heteropolysaccharide or a mixture of two different polysaccharides, it was then subjected to a Sephadex G50 column, which resulted in the elution of a single peak. These results strongly suggested the presence of a galactoglucan, although it is well known that polysaccharides that are able to complex by hydrogen bonds exist as double or triple helix conformation in aqueous solution [23,23]. Therefore we decided to treat the sample with SDS and repeat the elution by Sephadex G50. By this treatment, a major peak eluted, followed by a minor one. The monosaccharide composition of both peaks was evaluated by HPLC, and this showed that the first one consists of 97.6% of glucose, and a minor proportion of galactose, while the second one contains galactose (33.3%) and glucose (66.6%). We observed that the SDS treatment could separate the complex of polysaccharides, suggesting that the main molecule present in this extract is an α-glucan.

**Figure 3:**
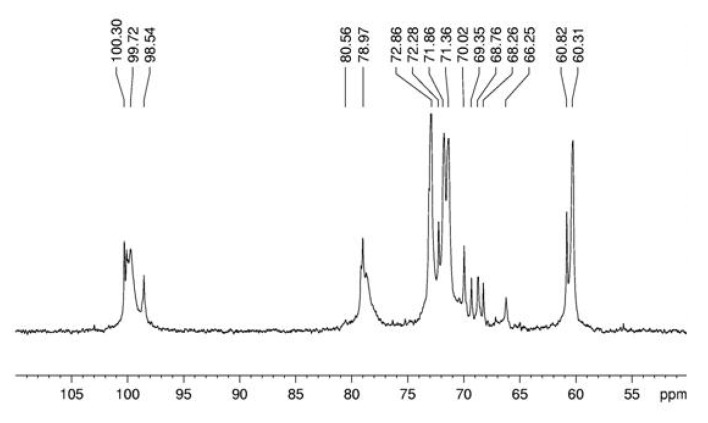
^13^C-NMR spectrum of α-glucan in dimethyl sulfoxide-*d_6_* at 70 °C (chemical shifts are expressed in δ ppm).

**Figure 4:**
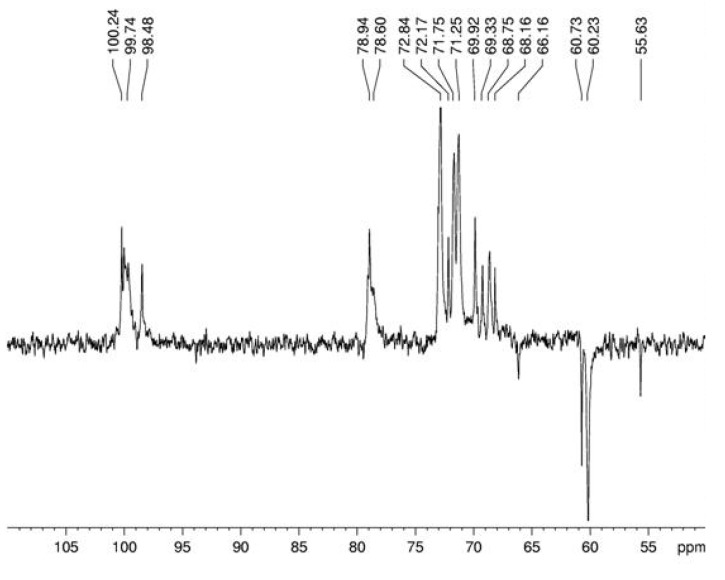
DEPT spectrum of α-glucan in dimethyl sulfoxide-*d_6_* at 70 °C (chemical shifts are expressed in δ ppm).

Spectroscopy experiments were performed in order to elucidate the linkage type of this polymer. ^13^C NMR (Figure 3) of this glucan showed signals in the anomeric region at δ 100.3; 100.1 and 99.7, characteristic of D-glucose in α configuration, and a sign at δ 98.5 probably arising from D-galactose in α configuration. The sign at δ 78.9 arises from O-4 substitution of Glc*p* units and it proves that the majority of the monosaccharide is (1→4)-linked [24]. The DEPT-^13^C-NMR (Figure 4) allowed assigning the non-substituted and *O*-substituted –CH_2_ signals at δ 60.3/60.8 and 66.2, respectively. The latter is an indicative of O-6 substitution, showing the presence of a branched glucan, confirming the data from methylation analysis [25]. Some more assignments were done as in the upmentioned study [24]: signals at δ 71.8; 72.8; 70.0; 71.3 and 60.3/60.8 correspond, respectively, to C-2, C-3, C-4, C-5 and C-6 of units A (Figure 6).

The main anomeric signals (C1/H1) in the HSQC spectrum (Figure 4) arose at δ 100.6/5.06; 100.2/5.11; and 98.8/4.74, corresponding to C-1 of B, C, and A units, respectively (Figure 6). The α-configuration of Glc*p* residues was confirmed by low frequency resonances of C-1 at δ 100.6, 100.2, and 98.8 and H-1 high field ones at δ 5.06, 5.11, and 4.74 [26]. From the above data, we conclude that the isolated polysaccharide (Figure 6) of *A. bisporus* is an α-glucan with a main chain consisting of (1→4)-linked units, substituted at O-6 by α-D-glucopyranose single-units in the relation 1:8. This molecule is similar to glycogen, a common fungal storage polysaccharide of which the degree of polymerization and the extent of branching can vary according to the intrinsic properties of the individual branching enzymes [27,28] available in the species. The total amount of this polymer is usually 5-10% of the dry matter, which seems to be nutritionally negligible [29]. As cited before, glycogen-like molecules are also able to enhance the production of NO by RAW264.7 cells into the culture fluid and promote the activation of macrophages, stimulating the TNF-α production and phagocytosis in the same cell line [10].

**Figure 5:**
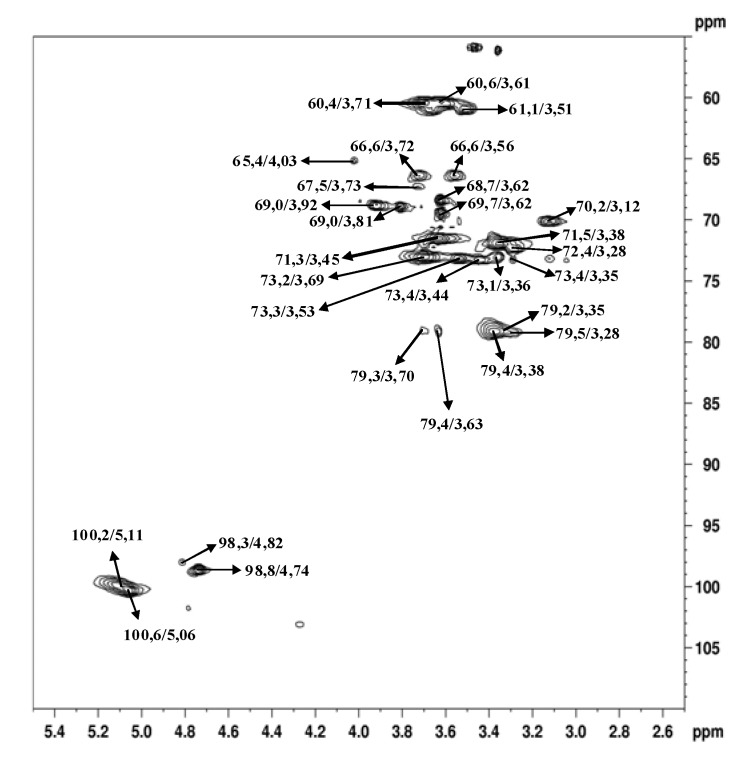
HSQC spectrum of α-glucan in dimethyl sulfoxide-*d_6_* at 70 °C (chemical shifts are expressed in δ ppm).

The immunomodulatory polysaccharides that have been described in most detail in fungi are the β-(1→3)-(1→6)-glucans [23], although also homo- and hetero-polysaccharides containing mannose, galactose, xylose, and fucose are found. Mannogalactans [30], xylomannans [31], and fucomannogalactans [32] have been well characterized from mushrooms of the genera *Pleurotus, Agaricus, Lentinus, Flammulina* and others. A galactoglucan was isolated from *Pleurotus sajor-caju* [33], presenting a main chain composed of α and β-D-Glc*p* alternating (1→4) and (1→6) linkages. This polymer is O-6 branched by α-D-Gal*p* units at each 3 units of the main chain and this is the only report about a galactoglucan isolated from mushrooms. A (1→3)-linked heterogalactan containing mainly galactose and small amounts of fucose was isolated from *Poria cocos* [34], and methyl-galactans (1→4)- and (1→6)-linked were isolated from *Pleurotus ostreatoroseus* and *Pleurotus eryngii* [35,36]. The presence of glycogen-like polymers in *Agaricus bisporus* has been reported before [37] but there is a lack of studies regarding the polysaccharides present in this species.

**Figure 6:**
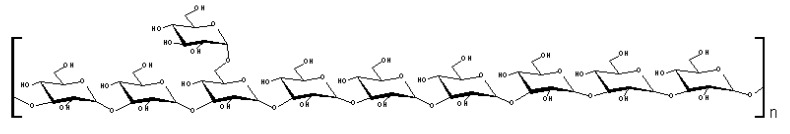
Structure of α-glucan isolated from *Agaricus bisporus*.

Our results suggests that the polysaccharide complexed with the α-glucan could be a low MW galactan, containing some methylgalactose, as demonstrated by the presence of the low intensity sign at δ 56.1 ppm arising from the CH_3_ group in the ^13^C-NMR spectrum [36]. Based on our earlier quantitative observations [15] we now estimate up to 90% of the hot water extracted polysaccharide of *A. bisporus* to consist of galactan linked α-glucan.

It has already been described by others [23] that polysaccharides can assume helical conformations in aqueous solution, and that this varies depending on the linkage type and the monosaccharide composition. It has been shown that schizophyllan adopts a triple helical conformation in water, but also has a random coil conformation in dimethylsulfoxide. This kind of complex is possible because of the hydrogen bonds that are formed by the polysaccharides [38].

There is a multitude of studies relating to the polysaccharides isolated from mushrooms and their biological activities. Different kinds of β-glucans, α-glucans, mannoglucans, galactomannans, fucogalactans, mannans, galactans, and also glycoproteins have been isolated from a variety of edible and non-edible mushrooms. Among the effects attributed to these molecules are the production of NO, cytokines as TNF-α, IL-1, IL-6, IL-8, and IL-12, and the stimulation of phagocytosis [23,39]. Specific proteins, terpenoids, steroids, fatty acids, and phenolic compounds were also shown to possess bioactive effects with, possibly, relevance for health homeostasis, such as immunomodulation, antihypertension, cytotoxic, antibacterial, and prooxidative effects, respectively [20,40]. Since all these compounds are found in mushrooms, and can produce differential effects on specific cells, the use of good techniques as NMR spectroscopy, methylation analysis, and treatment with pure enzymes are important to obtain well isolated and characterized extracts, that can be used to understand the mechanism of action of each of these compounds when tested *in vitro* or *in vivo*.

## 3. Experimental

### 3.1. Polysaccharides

Crude polysaccharide extracts were prepared from fruiting bodies of *Agaricus bisporus* strain Horst U1, obtained from Innerlife B.V. (Venlo, The Netherlands) by hot water extraction as described before (16) and concentrated to > 35 Brix for cold storage. Polysaccharides were semi-purified by precipitation with two volumes of 96% ethanol and repeated washing to remove the excess mannitol. Colored polysaccharide polyphenol complexes were adsorbed to DEAE cellulose and the non adsorbed polysaccharide was precipitated with two volumes of 96% ethanol, after which the adsorption procedure was repeated. The resulting purified polysaccharide precipitated as a white fibrous mass in 70% ethanol and was stored for further use in 70% ethanol.

### 3.2. General Experimental Procedures

All solutions were evaporated at < 40 °C under reduced pressure. Centrifugation was carried out at 8,000 rpm for 15 min, at 25 °C. Alditol acetate mixtures were analyzed by GC-MS using a Varian model 3300 gas chromatograph linked to a Finnigan Ion-Trap, model 810-R12 mass spectrometer, using a DB-225 capillary column (30 m × 0.25 mm i.d.) programmed from 50 to 220 °C at 40 °C/min, then hold. Partially *O*-methylated alditol acetate mixtures were similarly analyzed, but with a program from 50 to 215 °C at 40 °C/min. Anion exchange chromatography was performed on DEAE-cellulose with a linear gradient of 0.0–1.0 M NaCl in water as the mobile phase. Sephadex preparative size-exclusion chromatography was done on a water equilibrated Sephadex G50 column of 30 × 1.5 cm using a Pharmacia LKB FPLC system. DEAE–Cellulose D0909 and α-Amylase type XIIA from *Bacillus licheniformis* were from Sigma Chemical Corp (St. Louis, MO, USA).

### 3.3. Analyses and Determinations

#### 3.3.1. Carbohydrate Determination

Carbohydrate concentrations were determined by the phenol-sulphuric acid method, using D-glucose as the standard [40].

#### 3.3.2. Analysis of Monosaccharide Composition by GC-MS

Each polysaccharide fraction (1 mg) was hydrolyzed with 2 M TFA at 100 °C for 8 h, followed by evaporation to dryness. The dried carbohydrate samples were dissolved in 0.5 N NH_4_OH (100 µL), held at room temperature for 10–15 min in reinforced 4 mL Pyrex tubes with Teflon lined screw caps. NaBH_4_ (1 mg) was added, and the solution was maintained at 100 °C for 10 min, in order to reduce aldoses to alditols [42]. The product was dried and excess NaBH_4_ was neutralized by the addition of acetic acid or 1M TFA (100 µL), which was removed following the addition of methanol (× 2) under a N2 stream in a fume hood. Acetylation of the Me-alditols was performed in pyridine–Ac_2_O (200 µL; 1:1, v/v), heated for 30 min at 100 °C. The resulting alditol acetates were analyzed by GC-MS, and identified by their typical retention times and electron impact profiles. Gas liquid chromatography-mass spectrometry (GC-MS) was performed using a Varian (model 3300) gas chromatograph linked to a Finnigan Ion-Trap model 810 R-12 mass spectrometer, with He as carrier gas. A capillary column (30 m × 0.25 mm i.d.) of DB-225, held at 50 °C during injection and then programmed at 40 °C/min to 220 °C or 210 °C (constant temperature) was used for qualitative and quantitative analysis of alditol acetates and partially *O*-methylated alditol acetates, respectively [43].

#### 3.3.3. Analysis of Monosaccharide Composition by HPLC

The samples were hydrolyzed with 2 M TFA at 100 °C overnight, followed by evaporation to dryness. The residual TFA was removed by two evaporation cycles with 0.5 mL of MeOH, and the final residue was dissolved in 0.5 mL of H_2_O. After 100-fold dilution monosaccharides were determined using a Dionex HPLC system (Dionex Corp. Sunnyvale, Cal. USA) fitted with a Carbo Pac PA-1 column (4–250 mm), and a 25 μL sample loop with 20 mM NaOH isocratic solution (1 mL min^‑1^) as the mobile phase. An ED40 electrochemical detector fitted with a pulsed amperometric cell was used. Glucose and galactose were used as standards.

#### 3.3.4. Analysis of Sugar Oligomers

Purified polysaccharide was dissolved in 0.05 M PBS at pH 7.2 and incubated at 1 mg/mL with 300 U /mL *B. licheniformis* a-amylase for 1 h at 50 °C. The solution was then heated to 98 °C for 30 min and quenched on ice. After 100-fold dilution oligomers were determined using a Dionex HPLC system (Dionex Corp. Sunnyvale, Cal. USA) fitted with a Carbo Pac PA-1 column (4–250 mm), and a 25 μL sample loop. The columns were equilibrated with 125 mM NaOH. Sugars were eluted from the column using a isocratic gradient of 125 mM NaOH supplemented with 375 mM of sodium acetate (NaAc). Glucose and galactose were used as standards.

#### 3.3.5. Spectroscopy Analyses

Nuclear magnetic resonance (NMR) [13C and coupled 1H(obs.),13C heteronuclear single quantum correlation (HSQC) spectra] were obtained using a 400 MHz Bruker model DRX Avance spectrometer incorporating Fourier transform, as described before in detail [31]. Samples were dissolved in dimethyl sulfoxide-*d_6_* and examined at 70 °C. Chemical shifts are expressed in ppm (*δ*) relative to the resonance of dimethyl sulfoxide-*d_6_* at δ 39.70 (^13^C) and 2.40 (^1^H).

## 4. Conclusions

The results presented in this report show characterization of the major glucan of *A. bisporus* as a (1→4),(1→6)-α-glucan, which complexes with small polysaccharides as galactans by hydrogen bonding.
